# Preoperative multimodal imaging evaluation of a primary cardiac schwannoma of the right atrioventricular groove: a case report

**DOI:** 10.1093/ehjcr/ytaf238

**Published:** 2025-05-23

**Authors:** Yue Ren, Hongkai Zhang, Yao Lu, Rui Liu, Lei Xu

**Affiliations:** Department of Radiology, Beijing Anzhen Hospital, Beijing Institute of Heart, Lung, and Vascular Diseases, Capital Medical University, No. 2 Anzhen Road, Chaoyang District, Beijing 100029, China; Department of Radiology, Beijing Anzhen Hospital, Beijing Institute of Heart, Lung, and Vascular Diseases, Capital Medical University, No. 2 Anzhen Road, Chaoyang District, Beijing 100029, China; Department of Nuclear Medicine, Beijing Anzhen Hospital, Beijing Institute of Heart, Lung, and Vascular Diseases, Capital Medical University, No. 2 Anzhen Road, Chaoyang District, Beijing 100029, China; Department of Pathology, Beijing Anzhen Hospital, Beijing Institute of Heart, Lung, and Vascular Diseases, Capital Medical University, No. 2 Anzhen Road, Chaoyang District, Beijing 100029, China; Department of Radiology, Beijing Anzhen Hospital, Beijing Institute of Heart, Lung, and Vascular Diseases, Capital Medical University, No. 2 Anzhen Road, Chaoyang District, Beijing 100029, China

**Keywords:** Case report, Multimodal imaging, Cardiac tumour, Schwannoma

## Abstract

**Background:**

Cardiac schwannoma is exceedingly rare, and few literature reports are available. We reported a case of primary cardiac schwannoma that performed preoperative multimodal imaging, aiming to highlight the significance of multimodal imaging evaluation and deepen our understanding of this tumour.

**Case summary:**

A 66-year-old man presented to our hospital, as physical examination revealed the presence of a mediastinal mass over a month. Subsequently, the patient underwent comprehensive examination. The images demonstrated the mass compressed the right coronary artery, but no invasion. Resection of the tumour by surgery was conducted, and the patient remains asymptomatic at follow-up.

**Discussion:**

This case emphasizes the role of multimodal imaging in preoperative evaluation of such rare cardiac tumours. For benign cardiac schwannoma, the preferred treatment is surgical resection.

**Learning points**

- Combining imaging modalities enhances diagnostic accuracy by providing complementary information about tumour location, morphology, and relationship with critical structures, as well as ruling out malignancy or metastasis. This integrated approach is essential for guiding surgical planning.
- Histopathological examination remains the gold standard for confirming rare diagnoses like pericardial neurilemmomas, providing definitive insights into tumour nature and excluding other potential malignancies.

## Introduction

Primary cardiac tumours are extremely rare, with an incidence of only 0.001%–0.003%.^1^ Cardiac schwannoma is exceedingly rare, and few literature reports are available. Therefore, accurate identification of such tumours is of significance in developing treatment strategies and assessing the prognosis. Multimodal imaging evaluation is of immense value in diagnosis and differential diagnosis, evaluation of benign and malignant tumuors, and surgical decision. In this report, we describe a case of primary cardiac schwannoma, which was evaluated using transthoracic echocardiography, coronary computed tomography angiography (CCTA), and other modalities. Surgical treatment and management based on multimodal imaging have not been reported previously, and this report is expected to clarify the role of multimodal imaging evaluation for such patients.

## Summary figure

**Table:** 

Day 1	A 66-year-old male with asymptomatic mediastinal mass found during routine examination is admitted to our hospital.
Day 2	TTE revealed a mediastinal mass.
Day 4	CMR identified a mass in the right atrioventricular groove, detailing its composition, mobility, and relationship with the myocardium.
Day 6	CCTA showed compression of the right coronary artery without invasion.
Day 9	PET-CT indicated a hypermetabolic lesion in the mediastinum without distant metastasis.
Day 12	The patient underwent surgical resection, and the mass was completely excised without damaging the right coronary artery.
Day 15	Postoperative pathological examination confirmed the mass as a schwannoma.
Month 3	Follow-up TTE showed no signs of recurrence after discharge.

## Case presentation

A 66-year-old man presented to our hospital, as chest CT during the physical examination revealed the presence of a mediastinal tumour over a month. The patient had no obvious positive symptoms; however, he had a history of hypertension, hyperlipidaemia, diabetes, and coronary artery disease. Physical examination findings are unremarkable. Electrocardiogram showed a normal sinus rhythm, with left axis deviation and no ST-T wave changes. No specific laboratory findings were identified.

**Figure 1:**
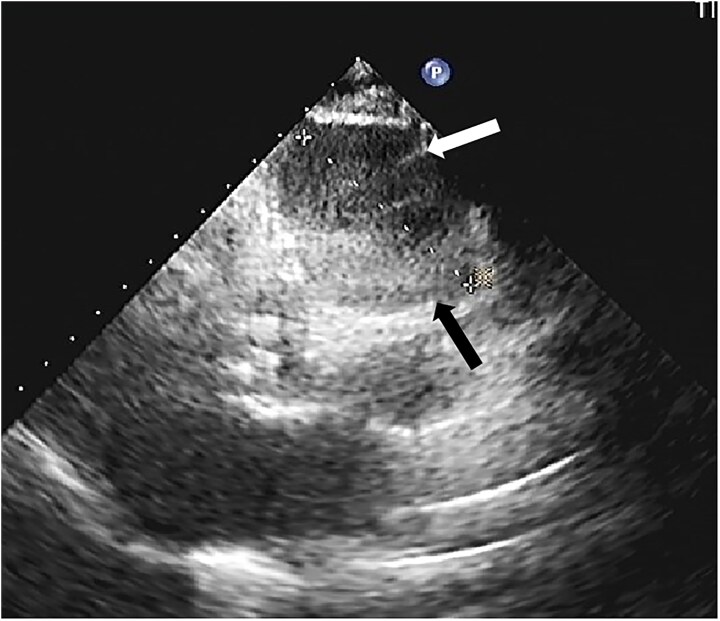
Transthoracic echocardiography revealed a tumour in the mediastinum (black arrow) with an anechoic area visible within the tumour (white arrow).

**Figure 2:**
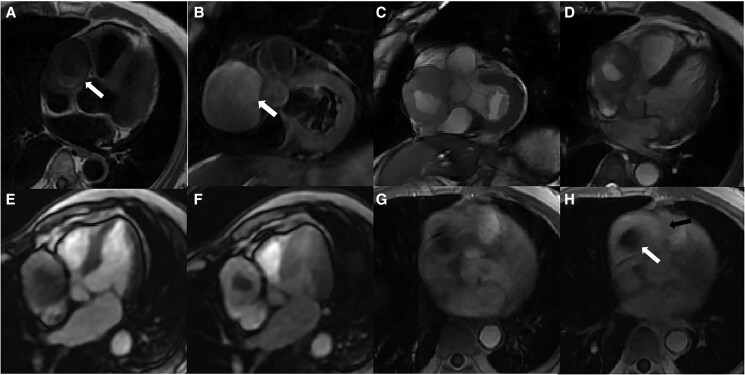
Cardiac magnetic resonance showing a mixed cystic and solid tumour compressing the right atrioventricular sulcus area. (*A*, *B*) The centre of the tumour presented low signal intensity, while the peripheral region shows a ring isointensity in axial T1-weighted imaging (*A*) (white arrow), but no signal reduction was observed in T2-weighted imaging fat suppression sequence (*B*) (white arrow). (*C*, *D*) The short-axis and four-chamber cine views showed T2-hyperintense the location of the tumour. (*E*, *F*) The solid part of the tumour was gradually enhanced in rest first-pass perfusion. (*G*, *H*) Early and late enhancement showed ring uniform enhancement of the solid components (white arrow), and no enhancement was observed in the central area of the tumour (black arrow).

**Figure 3:**
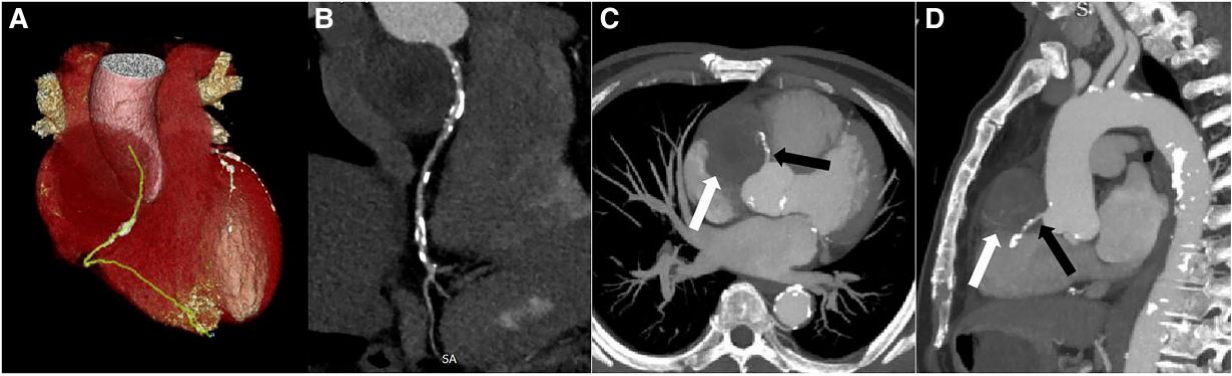
Coronary computed tomography angiography showed the tumour in the right atrioventricular sulcus area, adjacent to the pericardium and the right atrioventricular sulcus. (*A*) Volume rendering images provide a 3D visualization of the tumour located in the right atrioventricular groove. The line represents the right coronary artery. (*B*) Curved planar reformation images indicate that the right coronary artery follows a normal course. (*C*, *D*) Maximal intensity projection images demonstrate that the tumour compresses the proximal segment of the right coronary artery (black arrow: tumour; white arrow: compressed coronary artery).

**Figure 4:**
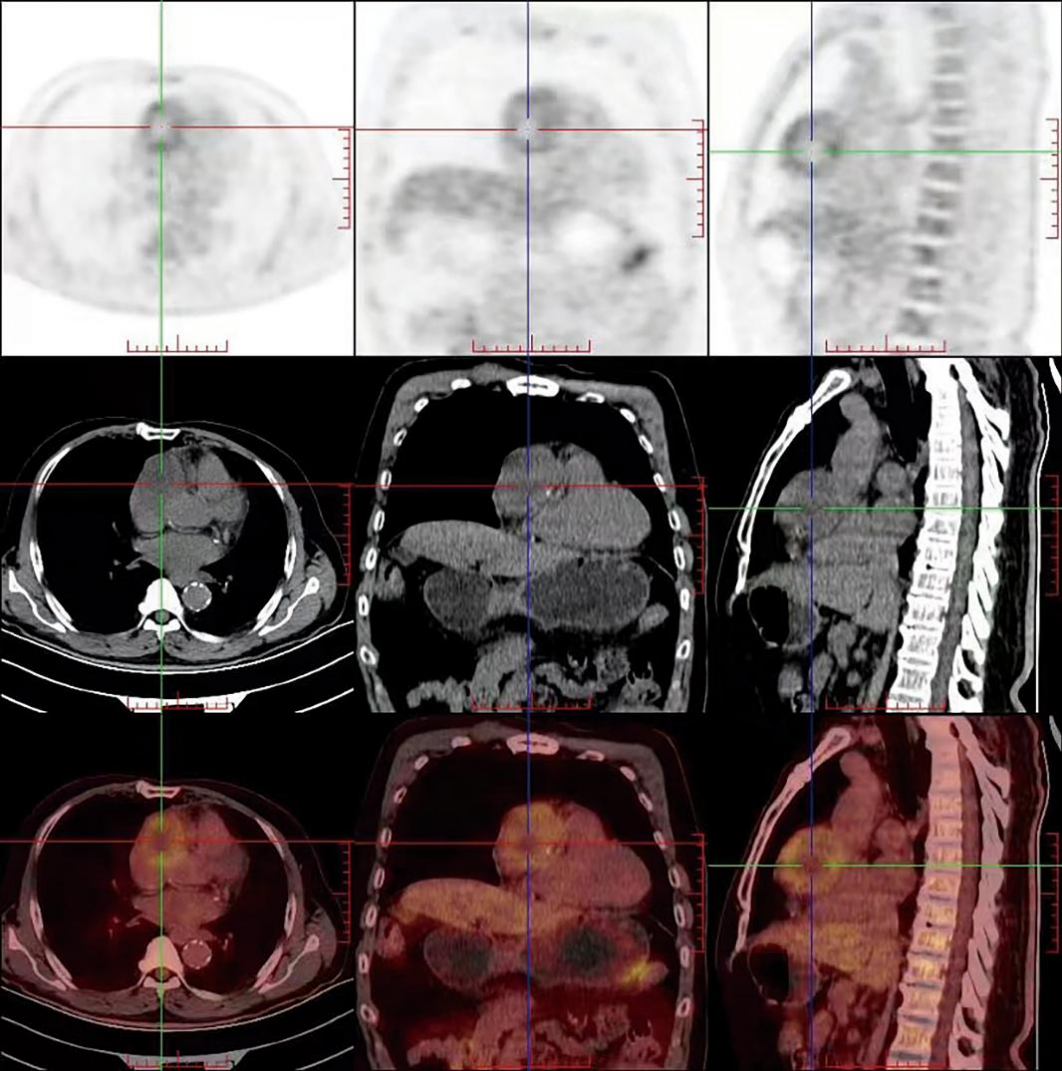
Positron emission tomography-computed tomography showing lesions with uneven ring-shaped increased uptake of fluorodeoxyglucose. The standard uptake value was ∼3.5.

As part of routine screening, chest X-ray and transthoracic echocardiography were initially performed. Chest X-ray revealed a tumour shadow along the right cardiac border, subsequently, echocardiography confirmed the presence of a large tumour (56 × 53 mm; *Figure 1*) in the mediastinum, with intact envelope, uneven internal echo, and clear boundaries. Cardiac magnetic resonance (CMR) and CCTA were performed subsequently. Cardiac magnetic resonance revealed the tumour located in the right atrioventricular groove, which obviously compressed the right atrium and ventricle. The tumour comprised a moderately enhancing component and a T2-hyperintense component in the centre without any enhancement (*Figure 2*). In addition, the tumour and the pericardium were not clearly demarcated; however, there were no gross myocardial infiltration and no tethering to the right atrium and ventricle. Coronary computed tomography angiography showed characteristics similar to the findings of CMR. Additionally, volume rendering, curved planar reformation, multi-planar reconstruction, and maximal intensity projection images clearly display the tumour’s position and its relationship with adjacent structures in multiple angles. The proximal segment of the right coronary artery is compressed by the tumour, but the vascular boundaries remain clear, with no obvious signs of invasion (*Figure 3*). To determine the presence of metastatic disease, fluorine 18 fluorodeoxyglucose (^18^F-FDG) positron emission tomography-CT (PET-CT) was performed, which revealed a mixed cystic and solid tumour with uneven ring-shaped increased uptake of FDG (*Figure 4*). The maximum standard uptake value (SUV) was ∼3.5. Obvious metastasis was not noted.

**Figure 5:**
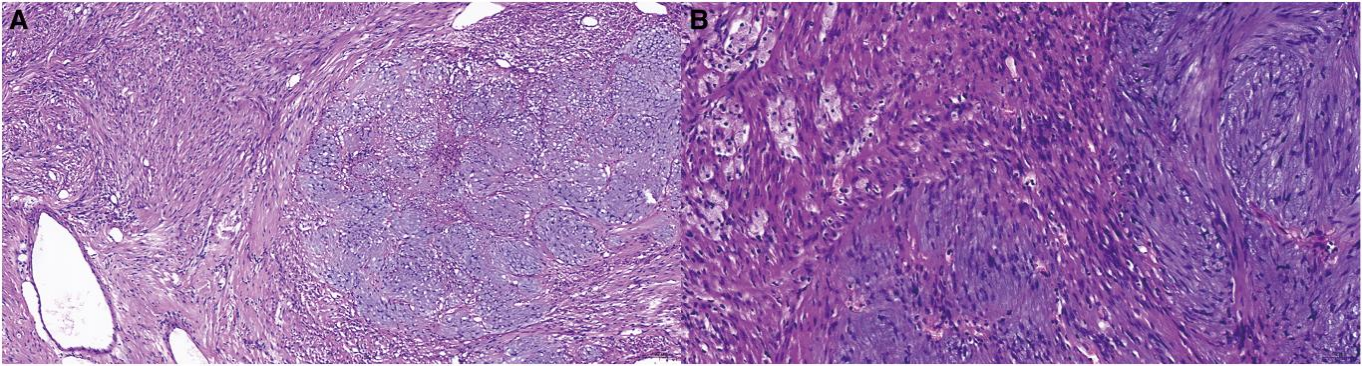
Histopathology revealing cardiac schwannoma consisting of spindle cells in a palisade-like arrangement. The magnifications of haematoxylin and eosin staining microscopy were 10 × 10 (*A*) and 10 × 20 (*B*).

The patient underwent thoracotomy and resection of the tumour under cardiopulmonary bypass to ensure adequate myocardial perfusion. During the surgery, care was taken to protect the surface blood vessels of the heart. The tumour was completely excised along the capsule without damaging the surrounding right coronary artery. The patient received postoperative dual antiplatelet therapy with aspirin and clopidogrel. Histopathological examination indicated that the excised tissue was a schwannoma (*Figure 5*). Microscopic sections revealed that the cells of Antoni A tissue had elongated, compact, and tapered nuclei, which were the characteristic findings. Immunohistochemistry showed that the tumour cells stained positively for S100 and SOX10.

Postoperative chest X-ray and aortic CCTA examination revealed that the tumour was completely resected without any residual tissue. The patient was discharged without any complications. At the 3-month follow-up, the patient recovered uneventfully, and echocardiography demonstrated preserved cardiac function with no significant evidence of recurrence.

## Discussion

Schwannoma originating in pericardium is rare, with previously reported tumour locations varying.^2–4^ In this case, the tumour was located in the right atrioventricular groove that has not been previously reported. Furthermore, the tumour in this case exhibited typical imaging characteristics, although no haemorrhage or calcification was observed. First, we considered the most common non-tumourous pericardial lesions. However, pericardial cysts and diverticula are typically composed of fluid, demonstrating homogeneous signal/density without enhancement.^5^ Next, we considered benign tumourous pericardial lesions. Fibromas are primarily composed of fibrous tissue and exhibit characteristic homogeneous high signal on late gadolinium enhancement. Teratomas are heterogeneous, often containing fat and calcifications. Lipomas are predominantly composed of fat and do not show enhancement.^1,6^ None of these features matched the findings in this case.

Multimodal imaging evaluation plays a vital role in the diagnostic workup of cardiac tumours.^7^ Chest X-ray is a primary screening tool that often reveals abnormalities such as cardiac silhouette enlargement or localized increased density, which may suggest the presence of a pericardial tumour. While it provides initial clues for further imaging evaluation, its diagnostic value is limited due to low resolution and poor tissue specificity. Echocardiography, as a non-invasive and real-time imaging modality, is crucial for evaluating pericardial lesions. It helps delineate the relationship between the tumour and cardiac chambers, valves, and pericardium, as well as detect pericardial effusion or cardiac compression.^5^ This is essential for assessing whether the tumour causes haemodynamic abnormalities and determining the urgency and necessity of surgical intervention. Because of its non-invasive and real-time nature, echocardiography is often used for the first-line diagnosis and long-term follow-up of cardiac tumours.^8^ Hence, this method was adopted in our case too. Cardiac magnetic resonance, as a one-stop examination, improves the comprehensive assessment of tumours and provides additional characteristic information of the myocardium simultaneously. Previously, Colin *et al*.^9^ reported a case of cardiac schwannoma; intraoperative exploration revealed that the tumour was closely related to the right ventricular outflow tract and pulmonary artery; hence, the attempt to resect the tumour failed. In our case, CMR was beneficial in discerning the positional relationship and in guiding the formulation of surgical strategies, compensating for the lack of information and reducing the likelihood of patients facing a secondary thoracotomy. In addition, CMR and echocardiography signified that the patient had good cardiac function, which laid the foundation for a successful surgery. Coronary computed tomography angiography, with its high resolution and advanced post-processing techniques, provides comprehensive visualization of the tumour's anatomical relationship with the coronary arteries. It helps confirm whether the tumour compresses the coronary arteries and affects myocardial perfusion, offering critical insights for surgical planning, including the potential need for vascular reconstruction or bypass grafting. In the management of cardiac tumours, the evaluation of malignancy and metastasis is crucial for patient risk stratification and selection of treatment strategies. Fluorine 18 fluorodeoxyglucose PET-CT is the most effective diagnostic tool for determining the nature of cardiac tumours.^10,11^ Previous experience with ¹⁸F-FDG PET-CT for pericardial schwannoma was limited, and the SUV was 3.5 (>2) in our case, which was suggestive of a malignant lesion. However, histopathological examination confirmed that it was a benign schwannoma. Inflammatory or infectious tumours can also present with a high uptake, which should be distinguished.^12,13^

Benign pericardial schwannomas exhibit biological behaviours similar to other benign tumours; therefore, the preferred therapeutic approach for this tumour is surgical resection.^2,14^ The gold standard for the diagnosis and classification of pericardial schwannoma is biopsy or histopathological examination, which was characterized by a variable admixture of Antoni A (compact spindled) areas and Antoni B (hypocellular and microcystic) areas.^15–17^ The histological features of hypercellular Antoni A areas and loose myxoid Antoni B areas correlate with imaging findings: Antoni A areas correspond to regions of marked enhancement on contrast-enhanced imaging, while Antoni B areas manifest as hyperintense signal on T2-weighted magnetic resonance imaging or hypodense regions on CT, reflecting the water content. This imaging-pathology concordance reinforces the diagnostic specificity of schwannoma and differentiates it from other pericardial tumours. Particularly, non-invasive comprehensive cardiovascular imaging may guide clinical treatment decisions and provide information on patient prognosis.^10^ In this case, we have reported a benign schwannoma that was subjected to a complete preoperative multimodal imaging evaluation and was subsequently resected successfully with a good prognosis.

## Lead author biography


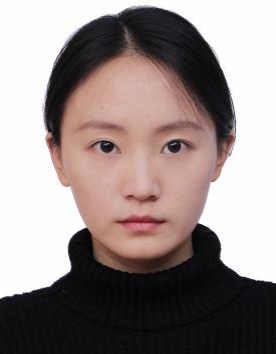


Yue Ren is currently a third-year student in the medical MSc programme at Capital Medical University in Beijing, China. Her research focus is on cardiovascular radiology.


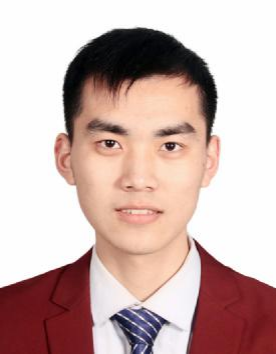


Hongkai Zhang is currently an attending radiologist at Beijing Anzhen Hospital in China, who has interest in medical cardiovascular imaging. He completed doctoral training at Capital Medical University in 2023.

*Consent:* The authors confirm that written consent for submission and publication of this case report including images and associated text has been received from the patient in line with the Committee on Publication Ethics (COPE) guidelines.

**Funding:** This work was supported by the National Key Research and Development Program of China (2022YFE0209800) and Beijing Natural Science Foundation (7244326).

## Data Availability

The data used during the current study are available from the corresponding author upon reasonable request.

## References

[1] Restrepo CS, Vargas D, Ocazionez D, Martínez-Jiménez S, Betancourt Cuellar SL, Gutierrez FR. Primary pericardial tumors. Radiographics 2013;33:1613–1630. 24108554 10.1148/rg.336135512

[2] Yun PJ, Huang TW, Li YF, Chang H, Lee SC, Kuo YL. Symptomatic pericardial schwannoma treated with video-assisted thoracic surgery: a case report. J Thorac Dis 2016;8:E349–E352. 27162698 10.21037/jtd.2016.03.40 PMC4842797

[3] Zhang XH, Wang Y, Quan XY, Liang B. Benign pericardial schwannoma in a Chinese woman: a case report. BMC Cardiovasc Disord 2013;13:45. 23800005 10.1186/1471-2261-13-45 PMC3699360

[4] Li C, Zhang J, Fan R, Chen L, Liu D, Lin H. Benign pericardial schwannoma with massive pericardial effusion in a Chinese man: a case report. Echocardiography 2019;36:1944–1946. 31654441 10.1111/echo.14492

[5] Rajiah P, Kanne JP, Kalahasti V, Schoenhagen P. Computed tomography of cardiac and pericardiac masses. J Cardiovasc Comput Tomogr 2011;5:16–29. 21051311 10.1016/j.jcct.2010.08.009

[6] Maleszewski JJ, Anavekar NS. Neoplastic pericardial disease. Cardiol Clin 2017;35:589–600. 29025549 10.1016/j.ccl.2017.07.011

[7] Yang Q, Liu H, Liu Y. Multimodal imaging changed clinical decision-making: a rare mediastinal yolk sac tumor infiltrating the heart and follow-up. Echocardiography 2021;38:1662–1665. 34435391 10.1111/echo.15180

[8] Lemasle M, Lavie Badie Y, Cariou E, Fournier P, Porterie J, Rousseau H, et al Contribution and performance of multimodal imaging in the diagnosis and management of cardiac masses. Int J Cardiovasc Imaging 2020;36:971–981. 32040684 10.1007/s10554-020-01774-z

[9] Colin GC, Gerber BL, Amzulescu M, Bogaert J. Cardiac myxoma: a contemporary multimodality imaging review. Int J Cardiovasc Imaging 2018;34:1789–1808. 29974293 10.1007/s10554-018-1396-z

[10] Aggeli C, Dimitroglou Y, Raftopoulos L, Sarri G, Mavrogeni S, Wong J, et al Cardiac masses: the role of cardiovascular imaging in the differential diagnosis. Diagnostics (Basel) 2020;10:1088. 33327646 10.3390/diagnostics10121088 PMC7765127

[11] Rahbar K, Seifarth H, Schäfers M, Stegger L, Hoffmeier A, Spieker T, et al Differentiation of malignant and benign cardiac tumors using ^18^F-FDG PET/CT. J Nucl Med 2012;53:856–863. 22577239 10.2967/jnumed.111.095364

[12] Colin GC, Vancraeynest D, Hoton D, Jonard P, Gerber B. Complete heart block caused by diffuse pseudotumoral cardiac involvement in granulomatosis with polyangiitis. Circulation 2015;132:e207–e210. 26503752 10.1161/CIRCULATIONAHA.115.017843

[13] Taskesen T, Goldberg SL, Mannelli L, Rabkin D, Hawn TR, Fligner CL, et al Granulomatosis with polyangiitis presenting with an intracardiac mass and complete heart block: enhanced images by 3-dimensional echocardiography. Circulation 2015;132:961–964. 26354785 10.1161/CIRCULATIONAHA.115.016851 PMC4976451

[14] Almobarak AA, AlShammari A, Alhomoudi RI, Eshaq AM, Algain SM, Jensen EC, et al Benign pericardial schwannoma: case report and summary of previously reported cases. Am J Case Rep 2018;19:90–94. 29362352 10.12659/AJCR.907408 PMC5789752

[15] Bussani R, Castrichini M, Restivo L, Fabris E, Porcari A, Ferro F, et al Cardiac tumors: diagnosis, prognosis, and treatment. Curr Cardiol Rep 2020;22:169. 33040219 10.1007/s11886-020-01420-z PMC7547967

[16] Tyebally S, Chen D, Bhattacharyya S, Mughrabi A, Hussain Z, Manisty C, et al Cardiac tumors: JACC CardioOncology state-of-the-art review. JACC CardioOncol 2020;2:293–311. 34396236 10.1016/j.jaccao.2020.05.009 PMC8352246

[17] Li S, Kusmirek JE, Buehler D, Kelly A, Schilling R, François C, et al A rare case of primary pericardial schwannoma. Radiol Cardiothorac Imaging 2021;3:e200176. 33778652 10.1148/ryct.2021200176 PMC7977734

